# Chemokine Homeostasis in Healthy Volunteers and during Pancreatic and Colorectal Tumor Growth in Murine Models

**DOI:** 10.3390/cimb44100339

**Published:** 2022-10-18

**Authors:** Elena V. Svirshchevskaya, Mariya V. Konovalova, Eugene V. Snezhkov, Rimma A. Poltavtseva, Sergey B. Akopov

**Affiliations:** 1 Shemyakin-Ovchinnikov Institute of Bioorganic Chemistry RAS, 16/10 Miklukho-Maklaya Str., 117997 Moscow, Russia; 2 National Medical Research Center of Obstetrics, Gynecology and Perinatology Named after Academician V. I. Kulakov of the Ministry of Health of the Russian Federation, 4 Oparina Str., 117997 Moscow, Russia

**Keywords:** chemokines, tumor model, CT26, Pan02, Luminex

## Abstract

Chemokines are involved in the humoral regulation of body homeostasis. Changes in the blood level of chemokines were found in cancer, atherosclerosis, diabetes, and other systemic diseases. It is essential to distinguish the effects of co-morbid pathologies and cancer on the level of chemokines in the blood. We aimed to analyze, by multiplex cytometry, the levels of chemokines in the blood of healthy young volunteers as well as of intact mice and mice with CT26 colon and Pan02 pancreatic tumors. Two types of chemokines were identified both in human and murine plasmas: homeostatic ones, which were found in high concentrations (>100 pg/mL), and inducible ones, which can be undetectable or determined at very low levels (0–100 pg/mL). There was a high variability in the chemokine levels, both in healthy humans and mice. To analyze chemokine levels during tumor growth, C57BL/6 and BALB/c were inoculated with Pan02 or CT26 tumor cells, accordingly. The tumors significantly differed in the growth and the mortality of mice. However, the blood chemokine levels did not change in tumor-bearing mice until the very late stages. Taken collectively, blood chemokine level is highly variable and reflects in situ homeostasis. Care should be taken when considering chemokines as prognostic parameters or therapeutic targets in cancer.

## 1. Introduction

Chemokines are 8–10 kDa polypeptides that bind to specific chemokine receptors and mediate the homeostatic regulation of tissues and the immune response. About 18–19 chemokine receptors and more than 50 chemokines binding to several receptors with different affinity are identified in humans. Table 1 summarizes the available data on the role of some chemokines in the formation of a tumor microenvironment.

Clones of macrophages and fibroblasts isolated from human bladder cancer tumors synthesize CXCL1 [1] in response to which neutrophils and other cell populations migrate to the tumor [2]. The role of CXCL1 and CXCR2 in osteosarcoma metastasis was shown [3]. The role of CXCL1 and CXCL5 in immunosuppression of infiltrating lymphocytes was demonstrated in the pancreatic cancer model [4]. In addition, the increased expression of CXCL1 is also associated with neoangiogenesis in the tumor [5]. The role of other CXCL chemokines is also shown in many works [6,7,8,9,10]. Chemokines play a role in the sensitivity to the checkpoint inhibitor therapy [11,12]. An increase in the level of CXCL9 was associated with tumor progression and an increased expression of CCR5 and CXCL13 was observed at the clonal level in patients who responded to the checkpoint therapy [12]. CXCL10 regulates migration and biological functions of CD4+ and CD8+ T cells in cancer and inflammatory autoimmune diseases [13,14,15,16].

The spectrum of activity of CCL chemokines is somewhat different. The involvement of CCL chemokines in the polarization of tumor-associated macrophages (TAMs) towards M2 is most often reported [17,18]. The involvement of CCL chemokines in different forms of cancer varies. Thus, the level of CCL2 is increased in breast, liver, and endometrial cancers and reduced in colorectal, lung, kidney, urothelial, and other cancers [19]. The situation is similar with other CCL chemokines [20]. More often, CCL2, 7, 8, 11, 13, and 26 were reduced in various forms of cancer, while CCL14, 17, 22, and 23 were increased [20].

It is well known that cancer often occurs in aging people, in whom co-morbid diseases are frequent, including atherosclerosis, diabetes, autoimmune diseases, and viral chronic infections. As an example, atherosclerosis is a disease where chemokines play roles both at early and ongoing stages [32,33,34,35]. Fractalkine (CX_3_CL1), GRO (CXCL1), MIF, CCL2, and CCL3 are involved in plaque initiation; CCL2, CCL3, CCL19, CCL21, CXCL9, CXCL10, CXCL11 CXCL12, and CXCL13 are crucial for the late stages of atherosclerosis [36,37,38]. Type I diabetes (T1D) is associated with an increase in Th1-associated chemokines such as IP-10 (CXCL10). Some papers also show an increase in Th2 chemokines CCL2 and CCL3 [39]. A wider range of chemokines (CCL2, CCL4, CCL5, CCL19, CCL22, CXCL9, CXCL10, CXCL11, and CXCL13) is secreted by purified human and mouse islets after stimulation with pro-inflammatory cytokines IFNγ or TNFα [39,40]. Multiple chemokines (CCL2, CCL3, CCL5, CX3CL1, CXCL8, CXCL9, CXCL10, CXCL11, and CXCL12) are increased in the blood of chronic obstructive pulmonary disease patients [41]. In chronic hepatitis C, the levels of CXCL9, CXCL10, and CXCL11 are also increased [42]. CCL5 is increased in herpes virus infection [43]. CXCL10 is considered a marker of rheumatoid arthritis [44]. Increased levels of CCL5 and CXCL6 are observed in bone degeneration patients [45]. Rotondi et al considered CXCL8 (IL-8) to play a major role in the formation of tumor microenvironment [46]. 

Due to a large number of chemokines regulating homeostasis, it is difficult to identify the most significant chemokine involved in tumor formation and response [19]. There may be several reasons for such differences: (1) different chemokines have the opposite effect on different types of tumors; (2) chemokines with different effects on different tumors do not directly affect tumor growth (satellites); (3) chemokines are involved in the regulation of homeostatic response to processes associated with co-morbid diseases; (4) the level of chemokines varies significantly between individuals. The latter makes it impossible to use chemokines as a prognostic factor in assessing the prognosis of tumor growth, as well as to develop drugs based on chemokine receptor inhibitors. In this paper, we tried to estimate chemokine levels in blood plasma of young healthy volunteers and understand the role of chemokines during tumor progression using CT26 mouse colorectal and Pan02 pancreatic cancer models.

## 2. Methods

### 2.1. Subjects and Ethics Statement

This study was carried out in accordance with recommendations from the local ethics committee in accordance with the Declaration of Helsinki. Blood samples were obtained from healthy volunteers of average age 28 ± 8 and different sexes (24 persons), who gave written informed consent prior to the study. Blood was collected in EDTA3 vacutainers, sedimented, plasma collected, frozen at −20 °C, and kept until use.

### 2.2. Mice and Ethics Statement

Female 6-to-8-week-old C57BL/6 and BALB/c mice were purchased from the Pushchino branch of the Shemyakin-Ovchinnikov Institute of Bioorganic Chemistry RAS and kept in the minimal disease conventional facility. All experiments were approved by the IBCh RAS Institutional Animal Committee. Protocols #259 (2018) and #325 (2021) were performed in compliance with AAALAC guidelines.

### 2.3. Multiplex Analysis of Chemokines

The standard 41-plex human cytokine–chemokine magnetic bead panel was used to analyze human chemokines in blood plasma by the FLEXMAP 3D cytometer (EMD Milipore, Billerica, MA, USA). Analysis was performed according to the manufacture instruction. In short, 25 µL of plasma was mixed with the beads in serum matrix provided by the manufacture. Before the analysis, plasma samples were cleared from insoluble material by centrifugation. Data were analyzed automatically with xPONENT software (EMD Millipore). The biolegend chemokine panel was used to analyze murine chemokines in blood plasma and cell supernatants (Biolegend, San Diego, CA, USA). Samples were run using the MACSQuant cytometer (Miltenyi Biotec, Bergisch Gladbach, Germany). The results were processed manually using FlowJo_V10 (FlowJo™, Ashland, OR, USA).

### 2.4. Tumor Models

Syngeneic subcutaneous tumor models were induced by the inoculation of 10^5^ CT26 (BALB/c) (*n* = 24) or Pan02 (C57BL/6) (*n* = 20) cells into the fur-depilated right flank in 100 µL of PBS. Tumor volumes were measured by the modified ellipsoidal formula V = (Length × Width^2^)/2 twice a week with an electronic caliper. Blood was collected from the eye orbital sinus under isofluran anesthesia and into heparin-treated tubes. Plasma was collected after centrifugation, frozen at −20 °C, and kept until use.

### 2.5. Histology

Fresh tumor tissues were fixed in 10% formalin and embedded in paraffin before sectioning and staining. Slides were prepared by a commercial firm (Pushchino, Moscow region, Russia). Tissue sections 4 μm thick were deparaffinized in xylene and rehydrated in ethanol series. The PicroSirius Red Stain Kit (Abcam, Cambridge, UK, ab150681) was used to stain connective tissue according to the recommended protocol. Masson’s trichrome (Chimmed, Moscow, Russian Federation) staining was used to stain collagen fibers and fibrin. Image acquisition was performed using a BHS system microscope (Olympus Corporation, Tokyo, Japan).

### 2.6. Statistical Analysis

Graphs were created using MS Excel software. The data are represented as mean ± SEM of at least three independent experiments or as one representative experiment from three. Statistical analysis was performed using Student’s *t*-test. Significance levels of *p* < 0.05 were considered statistically significant.

## 3. Results

### 3.1. Chemokines in Human Blood

**Figure 1:**
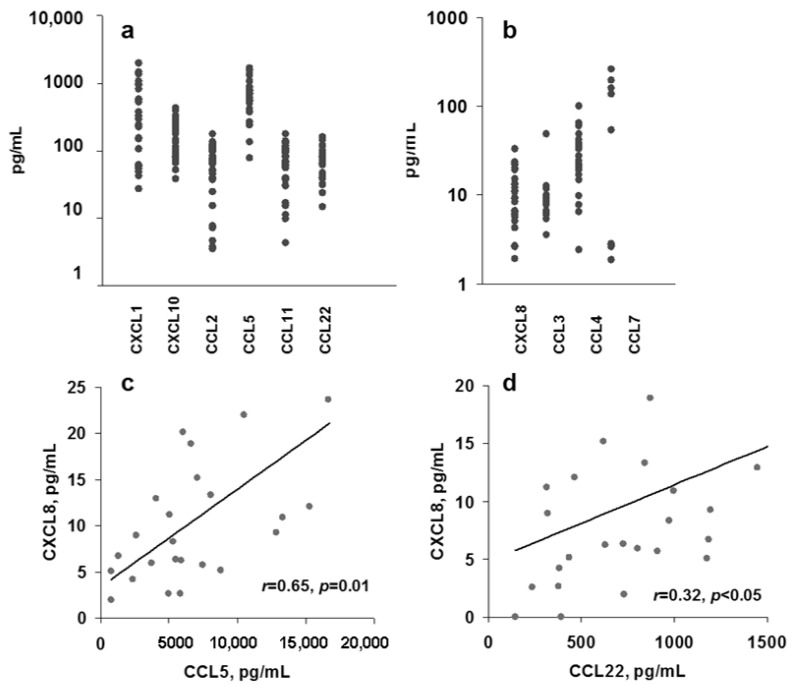
Chemokine levels in human blood plasma. (**a**,**b**) High (**a**) and low (**b**) chemokine levels (log scale) in blood plasma of 24 healthy volunteers. (**c**,**d**) Correlation between high (**c**) and low (**d**) level chemokines in donor plasma. Pearson’s coefficients of correlation and probabilities are shown (**c**,**d**).

Some chemokines are produced by constituently monitoring body homeostasis while others are likely to be inducible. We screened chemokine concentrations in blood plasma of 24 healthy young (average age 28 ± 8) volunteers. Among the 10 chemokines, tested CXCL1, CXCL10, CCL2, CCL5, CCL11, and CCL22 were found in each plasma at high levels (100–10,000 pg/mL, Figure 1a). This group is likely to serve homeostatic roles regulating body homeostasis (group I). Group II chemokines CXCL8 (IL-8), CCL3, CCL4, and CCL7 were detected at 0–300 pg/mL (Figure 1b) and are likely to be inducible as some plasma samples were completely negative. There was a high variability in the levels of all chemokines in different healthy people (Figure 1a,b). This difference can result from individual homeostatic characteristics. One possible reason for such variability could be a different sex hormone status. To obtain insight into this, we analyzed the chemokines in men and women. The results demonstrated that there were no differences between these groups (Figure S1).

High correlations were found between the average concentrations both for I and II groups of chemokines (*r* = 0.994, *p* < 0.001; *r* = 0.915, *p* < 0.05, accordingly). Other hormones can affect chemokine production, but this area needs additional studies.

The results on chemokine levels depend on the test system and cannot be directly compared. To verify the reliability of our results, we tested the same sets of plasma over two different days. The total concentration slightly varied, possibly due to the experimental errors in the titration of the standard samples. However, correlation of the results obtained by the same test system in different days was 0.998 (Figure S2).

Another possibility of chemokine variability in different people was temporary changes in the chemokine levels. To this end, the blood plasma of two donors was analyzed with 1 mo intervals. It appeared that there was a 1.2–2.2 times increase in the donor 1 parameters (Figure S3a) and a 1.4 to 2.9 decrease in the donor 2 parameters (Figure S3b). The results were obtained in a single measurement to avoid day-to-day variations in the test results. These results can mean a slow change in the chemokine levels; however, more studies should be conducted to ensure this trend as only the results from three people were obtained. Of note, the correlation between all chemokines was from 0.990 to 1 for both donors.

Other reasons for chemokine variability can be a response to infections, vessel conditions, as well as genetic differences. The latter is shown below in mice.

It was interesting to see whether some chemokines correlate. We found significant correlations between CCL5 and CCL22 with CXCL8 (IL-8) (Figure 1c,d). CCL5 also significantly correlated with CCL22 (not shown). CCL5 and CCL22 are group I chemokines, while CXCL8 is from group II. CCL5 and CCL22 interact with CCR4 expressed by innate immunity cells—dendritic ones and macrophages. IL-8 (CXCL8) is one of the main pro-inflammatory chemokines secreted by macrophages, epithelial, and endothelial cells. Its role also was shown in cancer [46].

The concentration of each chemokine in the blood of different donors differed significantly. The ratio of the maximum to minimum concentration was 18 for CXCL1, 10 for CCL5, and even 24 for CCL2.

### 3.2. Chemokines in Murine Blood

**Figure 2:**
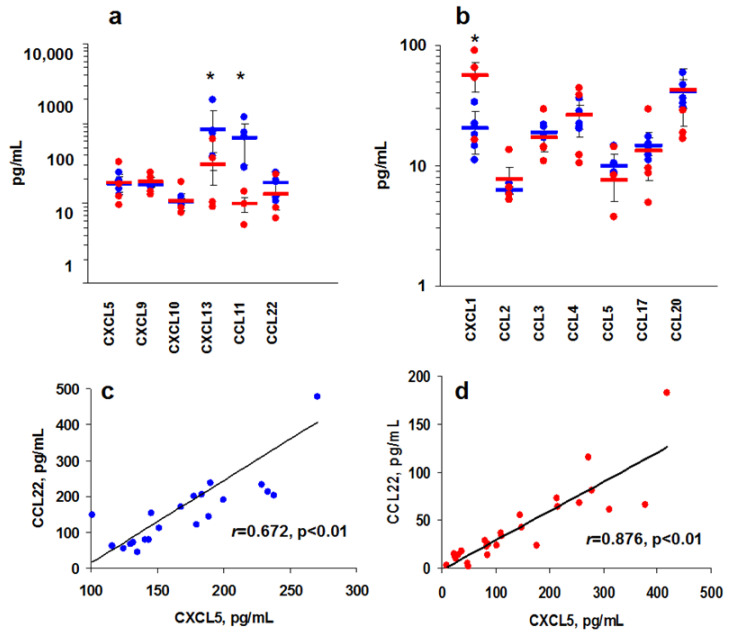
Chemokine levels in intact C57BL/6 and BALB/c blood plasma. (**a**,**b**): Variability of high (**a**) and low (**b**) chemokine levels in blood plasma of naïve C56BL/6 (*n* = 5) and BALB/c mice (*n* = 4). Significant (*t*-test, *: *p* < 0.05) statistical difference is shown with asterisks. (**c**,**d**): Correlation between CXCL5 and CCL22 levels in blood plasma from C57BL/6 (*n* = 21) (**c**) and BALB/c (*n* = 24) (**d**) mice. Pearson’s coefficients of correlation are shown. Significant difference between BALB/c and C57BL/7 mice is shown with the asterisks.

Among murine chemokines, high and low levels were also detected (Figure 2a,b). CXCL5, CXCL9, CXCL10, CXCL13, CCL11, and CCL22 were found at high levels (>100 pg/mL), while CXCL1, CCL2-5, CCL17, and CCL20 were found at low levels or were absent (<100 pg/mL) (Figure 2a,b).

We also found some correlations between chemokine levels. To this end, the whole pool of data (intact and tumor-bearing mice) was included (*n* = 44). There was a direct interdependence between CXCL5 and CCL22 both in C57BL/6 (Figure 2c) and BALB/c (Figure 2d) blood. Coefficients of correlation were higher for mice than for humans. There were also significant correlations between CXCL1 with CXCL5 and CCL22, as well as CXCL5 with CCL11 in BALB/c blood (Table S1). CXCL1 correlated with CCL22, CXCL5 with CXCL9 and CCL11, and CXCL13 with CCL22 in C57BL/c sera (Table S1). Correlation data both for human and murine chemokines show that the variability is specific for different individuals.

There was a difference between group I and II chemokines in human and murine blood. CXCL10, CCL11, and CCL22 were found at high concentrations, while CCL3 and CCl4 were found at low concentrations both in mice and humans (Table 2). CXCL1, CCL2, and CCL5 were found at high and low concentrations in human and murine blood, respectively.

### 3.3. Tumor Models

**Figure 3:**
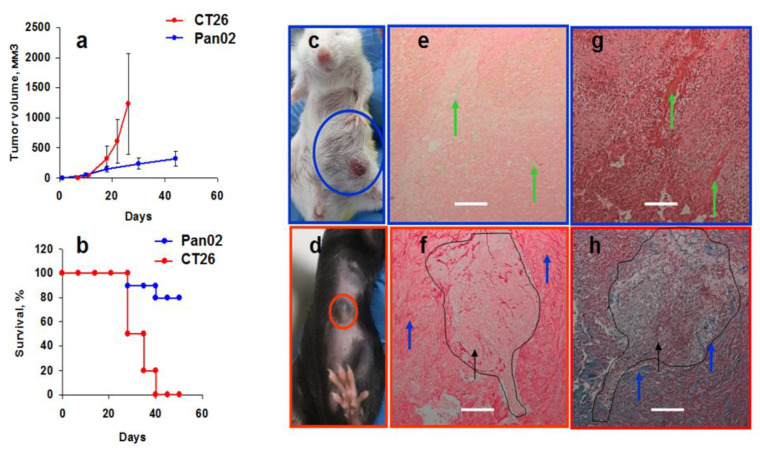
Pan02 and CT26 tumor growth characteristics. (**a**,**b**) In vivo tumor growth (**a**) and mice survival (**b**) in syngeneic CT26 (*n* = 24) and Pan02 (*n* = 24) models. (**c**,**d**): CT26 (**c**) and Pan02 (**d**) tumor sizes are shown with circles. (**e**–**h**) Histology of CT26 (**e**,**g**) and Pan02 (**f**,**h**) tumors stained by PicroSirius Red (**e**,**f**) or Masson trichrome (**g**,**h**) methods. Curves and black arrows mark epithelial cell growth among fibrotic matrix (blue arrows) in Pan02 tumors (**f**,**h**); green arrows show vessels in CT26 tumors. The experiments were repeated several times. Scale bar 200 µm.

Syngeneic tumor models were used to mimic human tumors. Based on the results of chemokines in intact murine blood, we tried to monitor chemokine changes during tumor growth. Subcutaneously transplanted pancreatic Pan02 and colorectal CT26 cells differed significantly in the in vivo tumor growth rate as was also shown earlier [47,48]. The volume of the Pan02 tumor in vivo never reached 500 mm^3^ and the mice survived longer, while the CT26 tumor grew exponentially, leading to mice deaths to days 40–50 (Figure 3a,b). Histological analysis showed that CT26 tumors were large, soft, non-fibrotic, and well enriched with blood vessels (Figure 3c,e,g). Pan02 tumors were small and extremely fibrotic with only a minor foci of tumor cells (Figure 3d,f,h). No signs of fibrosis in CT26 were found, as evidenced by PicroSirius Red (Figure 3e) or Masson trichrome (Figure 3g) staining, showing collagen containing connective tissue (red or blue, accordingly). A small number of vessels were found in Pan02 tumors. Evidently, Pan02 tumor cells stimulated extracellular matrix formation, including cancer-associated fibroblasts (CAFs), due to an insufficient blood supply limiting the tumor growth. The same high level of fibrosis is observed in human pancreatic adenocarcinomas [49].

New vessel formation requires signals from chemokines, including growth factors such as VEGF, FGF, PDGF, hypoxia-inducible factor HIF-1α, and cytokines [50]. Some chemokines stimulate angiogenesis, while others demonstrate angiostatic properties [51,52]. How the neovascularization and traffic of immune cells and CAFs to the tumor site relate to chemokine production is not well known. We compared chemokine levels in blood of intact and tumor-bearing mice.

### 3.4. Dynamics of Blood Chemokines in Syngeneic Pancreatic and Colorectal Tumor Models

**Figure 4:**
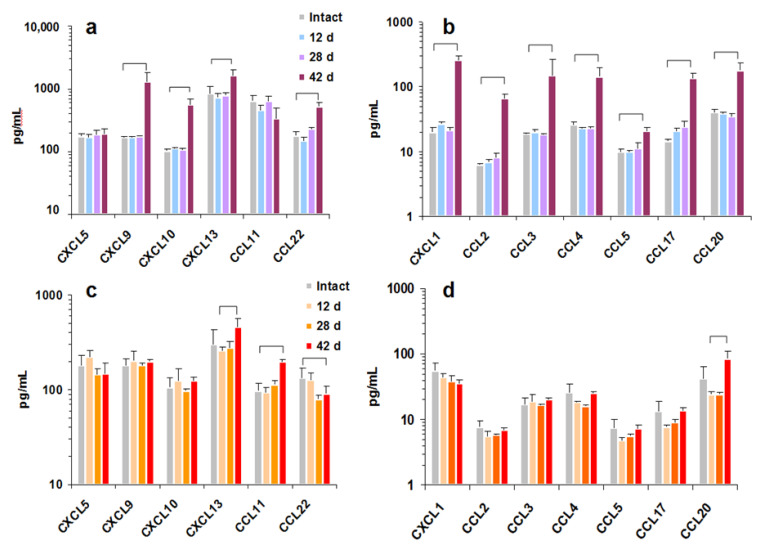
Chemokine levels during tumor growth. C57BL/6 (**a**,**b**) and BALB/c (**c**,**d**) were inoculated with syngeneic pancreatic tumor cells Pan02 (**a**,**b**) or colorectal cells CT26 (**c**,**d**) cells, respectively. Blood plasma was collected at days 0, 12, 28, and 42 following tumor inoculation. Group I (**a**,**c**) and II (**b**,**d**) chemokine levels are shown in the dynamics of tumor growth. Significant (*t*-test, *p* < 0.05) statistical difference is shown with brackets. The results are pooled from three independent experiments, while chemokine levels were measured simultaneously. Averages and SEMs are shown.

Blood chemokines were studied at days 0, 12, 28, and 42 following tumor inoculation. No changes were found at early stages of both tumor growths (Figure 4). On the 42nd day, multiple chemokines (CXCL1, 9, 13, CCL2, 3, 4, 20, and 22) were tremendously increased in C57BL/6 mice bearing pancreatic Pan02 tumors (Figure 4a,b). CXCL9 was increased 7.7 times, and CXCL10, CXCL1, CCL3, and CCL17 increased 5.5, 12.7, 10.6, and 8 times, accordingly. The only chemokine increased starting from day 12 in Pan02-bearing mice was CCL17 (TARC) (Figure 4b). A completely different pattern was found for CT26-bearing BALB/c mice. For the exception of CXCL13, CCL11, and CCL20, neither chemokine level changed (Figure 4c,d). CXCL13, CCL11, and CCL20 concentrations increased 1.5–2 times at day 42 versus intact mice; CCL22 decreased at days 28 and 42 (Figure 4c,d).

TARC (CCL17) is associated with neutrophil accumulation in tumors [53], the high infiltration of M2-like tumor-associated macrophages (TAMs) [54], the accumulation of fibroblasts [55], and a decreased traffic of Treg [56]. Taken collectively, an increase in CCL17 levels in Pan02 tumor-bearing mice shows the accumulation of innate immunity cells and fibroblasts forming significant extracellular matrix, as supported by the histological results.

## 4. Discussion

Chemokines play a role in wound healing, angiogenesis, and various other cellular functions including inflammation which directs the migration of immune cells to sites of infection. Earlier, it was shown that chemokines have both homeostatic and inflammatory functions [37,38]. Those that were found in blood at high levels evidently serve homeostatic purposes, while those found at low levels, such as IL-8 (CXCL8), are likely to be involved in inflammation or wound reparation. Multiple papers show that levels of many chemokines change in cancer patients. As cancer occurs mostly in aging people, it is important to consider the effects of co-morbid diseases on chemokine concentrations.

As demonstrated, chemokine levels vary significantly from person to person, even in young healthy people. This variability cannot be explained by the influence of the sex hormones, as we did not find differences between men and women. The same was also shown for mice. The levels of CXCL1, MIP-1α, and β were comparable in the blood of intact C57BL/6 male and female mice [57]. However, other hormones can play a role.

Murine data show that there is a difference in the chemokine profile of BALB/c and C57BL/6 mice, as CXCL13 and CCL11 concentrations were significantly higher in C57BL/6 mice than CXCL1 in BALB/c mice. These results can possibly indicate genetic variations; however, this area needs further study.

The data on chemokine levels in healthy people are limited. Differences in chemokine concentrations in the blood plasma of humans were shown by some authors. Nowak et al. analyzed chemokines CX3CL1 and CXCL12 in the blood of patients with ovarian cancer or benign ovarian tumors [58]. CX3CL1 was detected at 5–15 pg/mL, while CXCL12 was detected at 800–1000 pg/mL, coinciding with the inducible and homeostatic chemokines, respectively. The review by Cabrero-de Las Heras S and Martínez-Balibrea E summarized chemokines in blood of colorectal cancer patients [59]. They show high levels of CXCL1, CXCL7, CXCL10, and CXCL11, and low levels of CXCL8, coinciding with our data. Among them, only CXCL10 and CXCL8 had predictive values [59]. In our work, the ratio of the maximum to the minimum concentrations was 18 for CXCL1, 10 for CCL5, and even 24 for CCL2. When analyzing the potential prognostic significance of the changes in the level of chemokines in a group of cancer patients, overdiagnosis is likely to occur if these differences are not taken into account.

The role of chemokines and chemokine receptors in murine models of cancer is not well known. Analysis of multiple chemokines in the blood of mice without or with tumors is lacking. In another approach, the artificial regulation of some chemokines can affect tumor growth. For example, Liu et al. induced enhanced macrophage accumulation in the CT26 tumor by LPS and poly (I:C), associated with an increased CCL5 production, which resulted in accelerated tumor growth [60]. In the same tumor model, Kurzejamska et al. demonstrated that exogenously injected CCL7 also increased tumor progression [61]. An increase in CCL5 and CCL17 expressions stimulated by gene electrotransfer did not affect tumor growth [62]. Of note, CCL5, CCL7, and CCL17 are group II inducible chemokines. At the same time, the hyper-expression of CXCL10, a group I homeostatic chemokine, in the intraperitoneal CT26 model prevented liver metastasis [63]. All these and other works studied the role of chemokines by inducing their elevated levels either by gene over-expression or by exogenous proteins. This approach disturbs the homeostatic balance and may be not a proper model to study the effects of chemokines on tumor growth.

Our results show that chemokine levels do not change until the very late stages of both pancreatic and colorectal tumors. Song et al. did not find any differences in CCL2 and CCL5 levels in BALB/c mice with orthotopic mammary gland transplanted mice [64]. Ray et al. also did not register CXCL1, CCL3, and CCL4 changes in C57BL/6 mice bearing colon MC38 tumors [57]. There can be a difference in chemokine response to tumors in humans and mice. However, a huge variability in chemokine levels should be taken into account.

## 5. Conclusions

Chemokines are divided in two groups with high and low concentrations in the blood. It is likely that chemokines with high concentrations play homeostatic roles, while low ones are inducible. The level of both high and low chemokines vary significantly from person to person. Not only can cancer lead to a change in chemokine concentration, but also multiple co-morbid diseases often found in elderly patients. In the murine model of pancreatic and colorectal cancer, we did not find changes in chemokine levels at early stages of tumor progression. Taking into account a large number of chemokines, multiple receptors they interact with, interchangeability of multiple factors, and chemokine response to other co-morbid diseases, it is difficult to identify a single chemokine affecting tumor growth. Care should be taken when considering chemokine levels as a prognostic parameter or therapeutic target in cancer.

## Figures and Tables

**Table 1:** Role of chemokines in cancer.

Receptor	Chemokine	Effects in Tumor Microenvironment	Producers	Citations
CXCR2	CXCL1 (GRO, KC)	TANs, TIFs, TAMs, metastasis, macrophages, neutrophils, epithelial cells, Th17	Fibroblasts, epithelial cells, macrophages	[1,2,3,4,5]
CXCR2	CXCL5 (LIX)	TAMs, epithelial cells, metastasis, angiogenesis, epithelial–mesenchymal transition	Fibroblasts, epithelial cells	[6,7,8,9]
CXCR1,2	CXCL8 (IL-8)	Major inflammatory cytokine of innate immunity	Macrophages, epithelial, endothelial cells	[20]
CXCR3	CXCL10 (IP-10)	Th1, attracting CD8+ and CD4+ effector T cells to tumor sites; tumor-localized myeloid cells, including cDC1; sensitivity to PD1 therapy	Fibroblasts	[10,11,12,13,14,15]
CXCR5	CXCL13 (BLC)	B-cell recruitment, role in PD1 therapy	Lymphocytes, epithelial cells	[13,16]
CCR2, 4	CCL2 (MCP-1)	M1/M2 macrophage polarization; TIFs, metastasis, TAMs, Treg, Th17	Macrophages, fibroblasts	[17,18,19,21,22]
CCR1,4, 5	CCL3 (MIP-1α)	TAMs, TANs, osteoclast precursors	Macrophages	[23]
CCR1,5, 8	CCL4 (MIP-1β)	TAMs, TANs, osteoclast precursors	Macrophages, lymphocytes, epithelial cells	[24]
CCR1, 3, 4,5	CCL5 (RANTES)	Migration and recruitment of T cells, dendritic cells, eosinophils, NK cells, mast cells, and basophils	T-cells, monocytes, platelets, epithelial cells, fibroblasts	[25]
CCR3, 5	CCL11 (Eotaxin-1)	Eos, angiogenesis	Fibroblasts, epithelial cells, lymphocytes	[26]
CCR8	CCL17 (TARC)	Eos, TANs, TAMs, Treg, protection; progression, TILs, Th17, M1/M2 macrophage polarization	Macrophages	[27,28]
CCR6	CCL20 (MIP-3a)	Th17, Treg, angiogenesis	Macrophages	[29,30]
CCR4	CCL22 (MDC)	TILs, Treg, Th17, Eos, M1/M2 macrophage polarization, angiogenesis	Macrophages	[31]

**Table 2:** Comparison of group I and II chemokines in human and murine blood.

Receptor	CXCR2	CXCR2	CCR4	CCR4	CCR4	CCR4	CCR3	CCR4
	GRO(KC)	IP-10	MCP-1	MIP-1a	MIP-1b	Rantes	Eotaxin-1	MDC
	CXCL1	CXCL10	CCL2	CCL3	CCL4	CCL5	CCL11	CCL22
Human	high	high	high	low	low	high	high	high
Mice	low	high	low	low	low	low	high	high

## Supplementary Materials

The following supporting information can be downloaded at: https://www.mdpi.com/article/10.3390/cimb44100339/s1.

## Abbreviations

TANs	Tumor-associated neutrophils
TAMs	Tumor-associated macrophages
TILs	Tumor-infiltrating lymphocytes
TIFs	Tumor-infiltrations fibroblasts
Eos	Eosinophils
Treg	T regulatory cells
Th1, 2, 17	T-helpers 1, 2, 17
DC	Dendritic cells
M1/M2	Type 1 and 2 of differentiated macrophages
PD1	Programmed death receptor 1
CD4, 8	Cluster of differentiation
