# Supplementary material for: Chemokine Homeostasis in Healthy Volunteers and during Pancreatic and Colorectal Tumor Growth in Murine Models
Source: Curr Issues Mol Biol. 2022 Oct 18;44(10):4987–99. doi: 10.3390/cimb44100339 (PMC9600007; doi:10.3390/cimb44100339)
Supplement: Supplementary file 1 [file cimb-44-00339-s001.zip › cimb-1927714-supplementary.pdf]

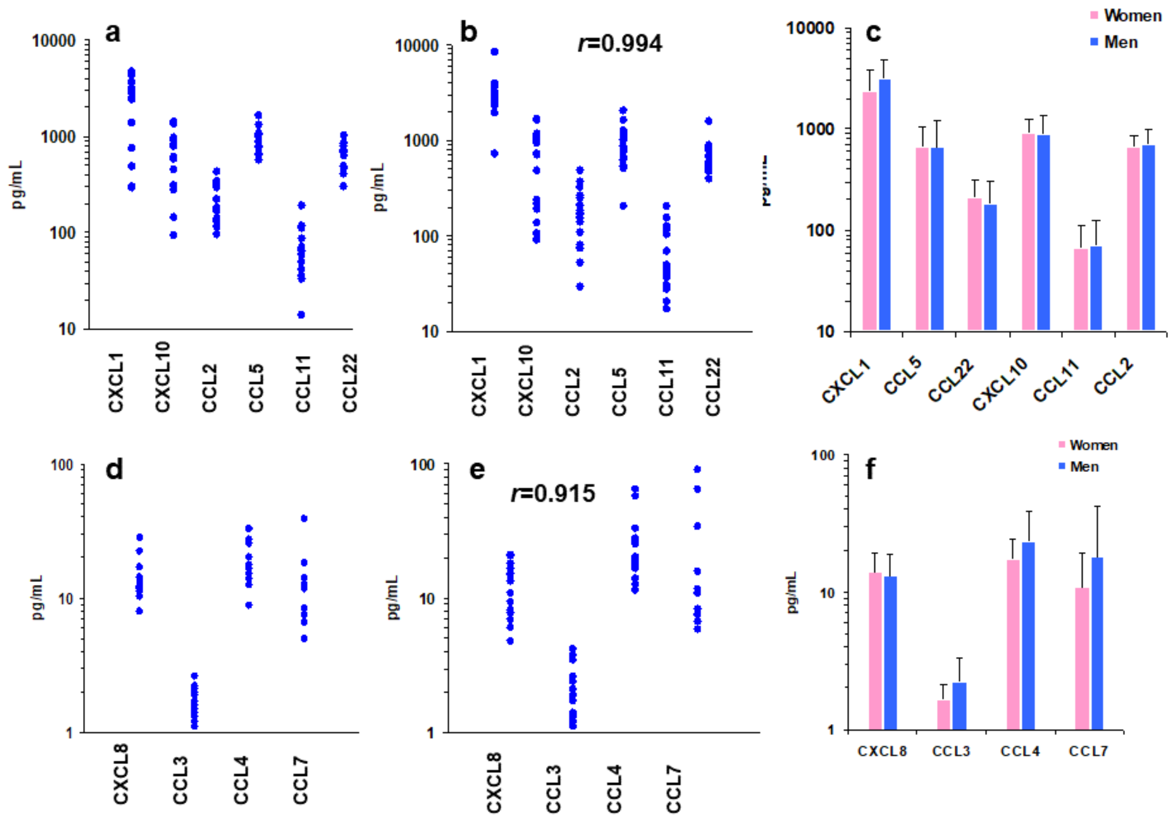

Figure S1. Individual (a, b, d, e) and average (c, f) chemokine levels in blood plasma of healthy young women (a, b) and men (d, e).

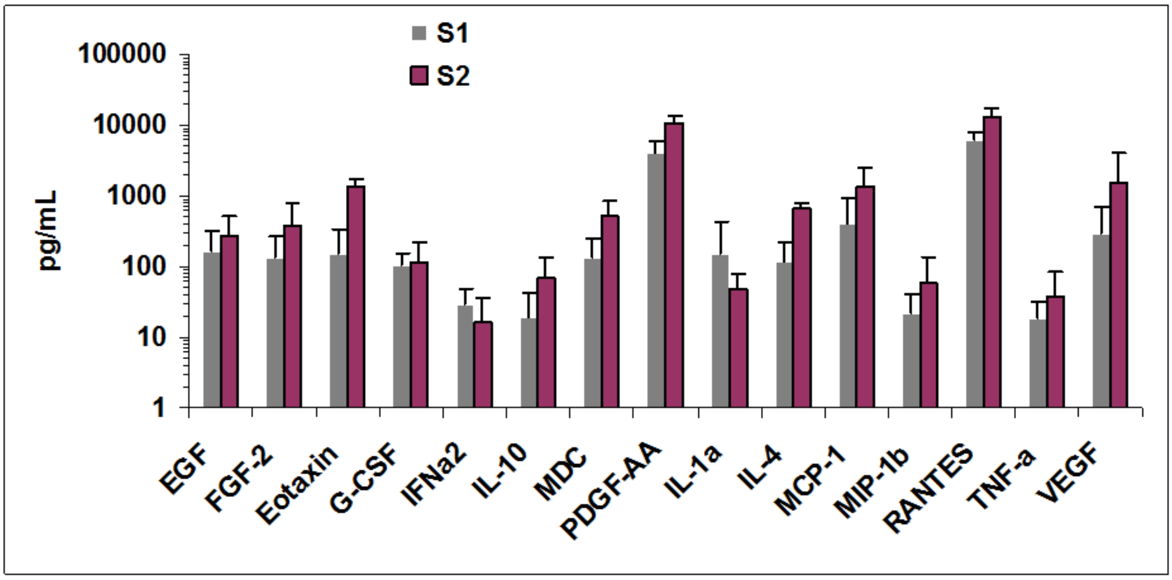

Figure S2. Analysis of the humoral factors in two independent measurements. Pearson's correlation coefficient was 0.988.

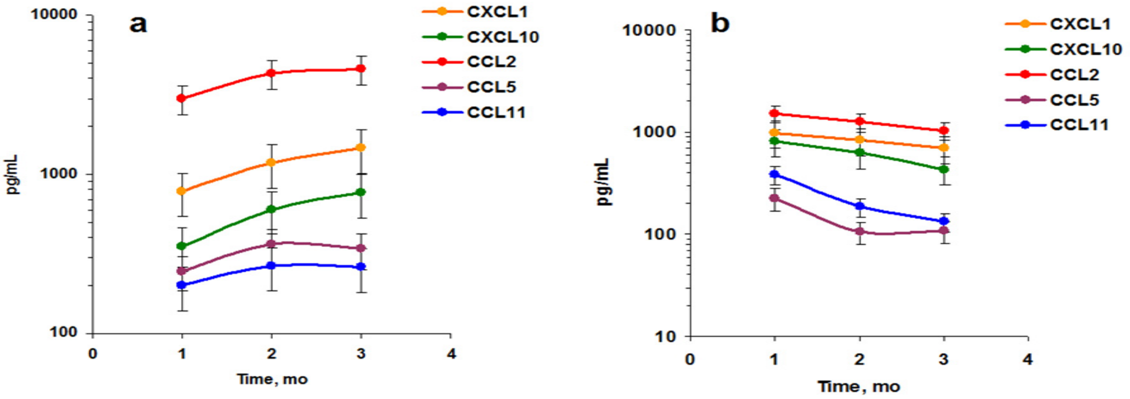

Figure S3. Changes in chemokine levels with time in two donors.

Table S1. Significant Pearson's correlation coefficients between the levels of chemokines in naïve C57BL/6 and BALB/c blood plasma

		CXCL1	CXCL5	CXCL9	CXCL13	CCL11	CCL22
C57BL/6	**CXCL1**	1					**0.33**
	**CXCL5**		1	**0.38**	**0.37**	**0.37**	**0.63**
	**CXCL9**			1	**0.37**		
	**CXCL13**				1		**0.44**
	**CCL11**					1	
	**CCL22**						1
BALB/c	**CXCL1**	1	**0.56**			**0.37**	**0.34**
	**CXCL5**		1				**0.88**
	**CXCL9**			1			
	**CXCL13**				1	**0.53**	
	**CCL11**					1	
	**CCL22**						1
